# Adipose-Derived Mesenchymal Stem Cells for the Treatment of Articular Cartilage: A Systematic Review on Preclinical and Clinical Evidence

**DOI:** 10.1155/2015/597652

**Published:** 2015-07-09

**Authors:** Francesco Perdisa, Natalia Gostyńska, Alice Roffi, Giuseppe Filardo, Maurilio Marcacci, Elizaveta Kon

**Affiliations:** ^1^II Clinic, Biomechanics Laboratory, Rizzoli Orthopaedic Institute, 40136 Bologna, Italy; ^2^II Clinic, Nano-Biotechnology Laboratory, Rizzoli Orthopaedic Institute, 40136 Bologna, Italy

## Abstract

Among the current therapeutic approaches for the regeneration of damaged articular cartilage, none has yet proven to offer results comparable to those of native hyaline cartilage. Recently, it has been claimed that the use of mesenchymal stem cells (MSCs) provides greater regenerative potential than differentiated cells, such as chondrocytes. Among the different kinds of MSCs available, adipose-derived mesenchymal stem cells (ADSCs) are emerging due to their abundancy and easiness to harvest. However, their mechanism of action and potential for cartilage regeneration are still under investigation, and many other aspects still need to be clarified. The aim of this systematic review is to give an overview of *in vivo* studies dealing with ADSCs, by summarizing the main evidence for the treatment of cartilage disease of the knee.

## 1. Introduction

Alterations in articular cartilage can produce pain and reduce the quality of life, evolving into the development of osteoarthritis (OA) and consequently permanent disabling symptoms.

Pharmacological or surgical treatments currently used for OA patients can help with the management of symptoms and delay disease progression [1–3], but metal resurfacing is the only currently available treatment able to provide pain relief and satisfactory function recovery, when the final stages of OA are reached [4–7]. Although new treatment options have come from recent achievements in biotechnologies [8, 9], the regeneration of hyaline cartilage is still a chimera [10]. Looking for a new direction in cartilage regeneration, the recent history of orthopaedics has been enriched with innovative therapies, ranging from platelet-derived growth factors (GFs) to cell-based treatments [11, 12], also combined with various biomaterials for tissue engineering strategies. In particular, recently mesenchymal stem cells (MSCs) are emerging as an alternative to the use of differentiated chondrocytes, thanks to their potential to differentiate into several lines such as osteoblasts, chondrocytes, myoblasts, or adipocytes and to their capability of self-renewal, high plasticity, and immunosuppressive and anti-inflammatory action [13]. MSCs can be obtained from different human sources, such as bone marrow, periosteum, umbilical cord blood, dermis, muscle, infrapatellar fat pad, synovial membrane, and adipose tissue [13]. Among these sources, adipose-derived mesenchymal stem cells (ADSCs) are attracting attention as an alternative to the better studied bone marrow mesenchymal stem cells (BMSCs) [14, 15]. The reasons for increased interest in ADSCs reside in their abundance (ADSCs are 5% of nucleated cells versus 0.0001–0.01% of BMSCs), the ease with which they can be harvested (with the advantages of lower donor-site morbidity), and their rapid expansion and high proliferation potential [16]; moreover, they have shown that they can maintain their phenotype better over many culture passages with respect to BMSCs [17]. Thus, over the last decade, the number of preclinical and clinical papers dealing with ADSCs has increased significantly. However, their mechanism of action is not fully understood, since both the differentiation of stem cells by themselves and paracrine and trophic effects might be involved. Moreover, the optimal strategy for applying ADSCs has not yet been identified and many aspects still remain controversial [18].

The aim of this systematic review is to analyze the current literature on the use of ADSCs* in vivo* to show the available evidence on their therapeutic potential for cartilage regeneration, by investigating their efficacy, drawbacks, and possible future application strategies in humans for the treatment of chondral pathologies.

## 2. Materials and Methods

A systematic review of the literature was performed on the use of ADSCs for knee cartilage defects* in vivo*. The search was made using the PubMed database, by focusing on both preclinical and clinical studies, with the following thread: “cartilage” AND “adipose-derived mesenchymal stem cells” OR “adipose-derived stem cells” OR “adipose derived stromal cells” OR “stromal vascular fraction.” The filters included publications from the last 10 years in the English language. Articles were first screened by title and abstract. Subsequently, the full texts of the resulting articles were screened and those not reporting the* in vivo* use of ADSCs or applications different from knee cartilage defects or OA were excluded. Cell sources other than adipose tissue were rejected unless they were used for comparative studies. All the articles dealing with applications of ADSCs different from those described above were also excluded. Reference lists of the selected articles were also screened to obtain further studies for this review.

## 3. Results

Following the above-mentioned screening procedure, 361 articles were initially found. Thirty-nine studies, meeting the above-mentioned criteria, were included in the final analysis: 28 animal studies (25 preclinical and 3 clinical) and 11 clinical studies. Among preclinical studies, 16 described arthrotomic implantation as a surgical procedure to treat focal osteochondral defects and 8 studies described intra-articular injections for an OA model. A further study evaluated 2 treatment groups for induced OA: by either injective or surgical administration. Culture-expanded ADSCs were used in all studies except one, where cells were isolated from adipose tissue by enzymatic digestion. A single study compared both methods. In 4 cases, expanded cells were manipulated by introducing different genes.

Among the clinical studies, 11 papers dealing with the administration of cells obtained from adipose tissue for the treatment of knee articular cartilage were included. Only one study described expanded ADSCs in the clinical setting, whereas all other cases reported on cells isolated by the enzymatic digestion of fat tissue.

The articles are described in detail in Tables 1 and 2, and the different aspects evaluated are summarized and discussed in the following paragraph.

## 4. Discussion

Over the last decade, an increasing number of papers have been published on the use of ADSCs for the treatment of cartilage disease, ranging from preclinical studies to clinical trials in the most recent years. In fact, adipose tissue is an appealing source of MSCs in the clinical setting, being an easy and abundant source for their harvest, which can be performed through a minimally invasive procedure, thus allowing a large number of cells to be obtained with minimal manipulations.

The studies analyzed in this systematic review present a variety of applications: cells can be used as a suspension after being cultured or freshly isolated by the enzyme-digestion of adipose tissue, thus obtaining a heterogeneous “stromal vascular fraction” (SVF) that contains a variety of stem, progenitor, and adult cells. Among them, it has been shown that ADSCs represent up to 10% of SVF [12]. The delivery can be performed by intra-articular injections, where cells are often suspended in a volume of platelet-rich plasma (PRP), or by surgical implantation, possibly by seeding them onto biomaterials, in the form of three-dimensional scaffolds.

Among the several papers analyzed, one of the first aspects to be underlined is the discrepancy between the approach used for their administration in the preclinical model and the modality actually predominant in the clinical setting. Preclinical trials used different animal models (16 rabbits, 3 mice, 1 rat, 2 pigs, 1 goat, 1 horse, and 1 sheep) and either expanded ADSCs (23) or SVF (1) were applied, plus 1 study that compared both methods. Cells were chondrogenic-induced (5) or noninduced (20), treated with GFs derived from platelets (2), or manipulated by introducing inductive genes (2). Conversely, the literature shows a different trend in the clinical application, both in terms of cell processing and delivery techniques: in fact, enzyme-isolated cells, in the form of SVF, were used in 13 of the 14 clinical studies (3 dog and 11 human), probably due to ease of manipulation and lower costs of a one-step procedure. This relies on the relatively high number of cells obtained from fat tissue with respect to other sources. Moreover, in all except one clinical study, cells were administered by intra-articular injection, whereas only one study reports the results of SVF surgical implantation, using fibrin glue as a scaffold for the treatment of isolated lesions. In fact, injective administration presents several advantages: it is minimally invasive, it has better patient compliance, and costs are lower [19]. Moreover, the rationale of this approach is to target not only the articular cartilage but also the whole joint environment, which is likely to be involved especially in degenerative diseases like OA [20, 21]. Most of the studies described cell delivery following arthroscopic debridement and aimed at maximizing the benefits of both approaches.

## 5. Preclinical Application

Since the preclinical setting allows researchers to focus better on specific issues, some interesting findings emerged with regard to the different aspects of using ADSCs. In the following paragraphs, we have summarized the main evidence and indications that emerged from the systematic literature analysis.

### 5.1. Mechanism of Action

The regenerative effect of ADSCs was suggested by Nathan and colleagues, who used ADSCs seeded onto a fibrin scaffold for the treatment of osteochondral defects in a rabbit model [22]. Better reconstruction was observed using allogeneic ADSCs compared to using cells from periosteum or a control group [22]. Conversely, three different studies showed poorer outcomes for ADSCs compared to GFs or other cell types in the rabbit model. Im and Lee reported better macroscopic scores by the sole application of GFs (TGF-b2 and BMP-7), with an uncertain effect of adding ADSCs [23], when treating osteochondral defects in rabbits. With regard to cell source, Koga et al. also showed lower cartilage matrix production and lower chondrogenic potential for ADSCs compared with BMSCs and synovial cells [24]. Similar findings were confirmed by Li et al., who showed the best results for BMSCs in terms of matrix production [25], and more recently by Ude et al., who reported better chondrogenic induction and gene expressions for BMSCs compared to ADSCs [26]. However, no differences were observed in terms of quality of cartilage regeneration in an induced-OA animal model [26]. Frisbie and colleagues reported that injecting cultured cells produces a comparable clinical, radiological, histological, and biochemical improvement, using either BMSC or ADSCs, for the treatment of induced OA in a horse model. However, the authors did not observe significant treatment effects to recommend stem cell treatment for this specific model of OA [27].

All studies except one [27] reported that the administration of ADSCs had a regenerative effect on articular cartilage in the animal model. However, the mechanism of action still remains unclear. Considering which cell may be the best progenitor for hyaline cartilage is still a subject of study [18]; currently, the leading hypothesis is that trophic activity plays a more important role than the intrinsic differentiation potential in the mechanism of action of ADSCs [18, 28], but future studies are needed to clarify this key aspect of ADSCs.

### 5.2. To Expand or Not to Expand?

All except two preclinical trials tested culture-expanded ADSCs and aimed at combining the greatest number of stem cells with various biomaterials for the treatment of critical-size osteochondral defects in the animal model. Two studies used ADSCs in the form of SVF, isolated by the enzymatic digestion of adipose tissue [29, 30]. van Pham et al. injected ADSC-SVF, previously pretreated with PRP, as a therapy for cartilage injury in mice, which showed improved joint regeneration and the lack of adverse events [30]. Jurgens and colleagues then highlighted the safety and feasibility of this approach by using freshly isolated SVF for one-step osteochondral repair in a goat model [31]. A comparison with expanded ADSCs showed no significant differences between the procedures: after slightly better scores for cultured ADSCs at 4 weeks, the SVF group proceeded towards better cartilage regeneration at the final evaluation. The authors argue that nonexpanded cells might have higher differentiation potential than expanded ones, exploited by a sort of promoting effect on the regenerative process of the other cell types contained in SVF [31].

Certainly, the use of cultured cells allows researchers to isolate and better characterize the desired cell type; however, economic and regulatory issues favor minimal manipulation procedures in the clinical practice. At the present time, the available preclinical literature shows no substantial evidence in favor of any of the two methods, and comparative studies with specific focus are required.

### 5.3. Injective Therapies for OA: How and When?

All 9 preclinical studies dealing with animal OA models reported a positive effect for the intra-articular delivery of ADSCs, with better improvement in the quality of the cartilage with respect to control or sham groups, regardless of the animal model. Toghraie et al. showed the efficacy of injecting ADSCs for induced-OA treatment in a rabbit model [32, 33]. The effect of a single injection produced significant improvement in cartilage and subchondral bone features, thus delaying the progression of OA, regardless of the source of harvest (infrapatellar fat pad [32] or subcutaneous adipose tissue [33]). Similar findings were observed by Ude et al., who observed regenerative features after a single-dose injection [26], either using induced ADSCs or BMSCs, for the treatment of induced OA in a sheep model. The inhibitory effect of ADSCs on the early stages of OA was confirmed by Ter Huurne and colleagues, who injected cells previously marked with green fluorescent protein (GFP) in a mice model. Interestingly, no effect was observed in late stages of OA [34]. Finally, Desando et al. found that injecting a lower dose of cells (2 × 10^6^) has more beneficial effects than a higher dose (6 × 10^6^) on OA progression, especially in earlier phases of the disease (16 versus 24 weeks) [35]. Thus, both dose and timing of ADSC administration should be considered, even though further studies are needed for a better definition.

### 5.4. Focal Osteochondral Defect Treatment: Surgical Implantation

Sixteen studies reported the use of ADSCs to treat osteochondral defects in different animal models. Adipose tissue was harvested from subcutaneous tissue in different sites. ADSCs were implanted into the lesions in combination with various biomaterials (ranging from fibrin glue [36] to collagen type I/III matrixes [31], PGA/PLA alone [37, 38] or polymers with chitosan [39], Ca-alginate [40], atelocollagen honeycomb-shaped scaffold with a membrane sealing (ACHMS) [41], polycaprolactone (PCL)/F127 [23], demineralized bone matrix (DBM) [25], or poly(L-lactic-co-glycolic acid) [42]).

Generally, improved tissue regeneration has been highlighted by seeding ADSCs into scaffolds, with good defect repair and the lack of adverse reactions, regardless of the type of biomaterial implanted. Unfortunately, the different biomaterials used and the variability of the study designs prevent any comparison among them.

In conclusion, each technique has shown that it can produce improved outcomes when applied to osteochondral lesions in the animal model, but comparative studies are lacking, and there is no evidence concerning the best treatment modality and the optimal biomaterial to be combined with ADSCs for cartilage regeneration.

### 5.5. Low Chondrogenic Potential of ADSCs and Improvement Strategies

Some authors tried to overcome the above-mentioned relatively low chondrogenic potential of ADSCs with different approaches. Shi and colleagues showed that BMP-4 plasmid transfected cells improved chondrogenesis in a PLLGA scaffold in a rabbit model, with respect to nontransfected ADSCs [42]. Lee and Im had similar findings by combining fibrin gel scaffold and cells transduced with SOX-trio proteins in a rat model [43].

Other studies showed the effect of platelet-derived GFs. Xie and colleagues implanted a PRP scaffold seeded with ADSCs or BMSCs in a rabbit model [44]: although BMSCs produced better tissue quality, good outcomes were observed using both cell lines, by having an inductive effect of PRP on MSCs proliferation and cartilage production [44]. Similarly, two studies by van Pham and colleagues tested the administration of human ADSCs, either expanded [30] or isolated as SVF [29], in a mouse OA model. No adverse reactions from the xenogeneic transplant were observed. Moreover, ADSCs pretreated with platelet-derived GFs showed a tendency towards better regeneration in both studies, with no differences between expanded ADSCs or SVF [29, 30]. Finally, one study showed the best defect filling and integration by combining GFs with predifferentiated ADSCs, among other groups with nonpredifferentiated cells or chondrocytes [45].

In conclusion, the regenerative effect of ADSCs can be induced in several ways. The combination of GFs can be a direct and easy method to improve the regenerative cell potential. On the other hand, manipulating ADSCs by introducing inductive genes has also shown improved cell features. Thus, further studies are necessary to determine which method is more effective and whether their combination may be beneficial.

## 6. Clinical Findings

Three clinical animal studies were found, all reporting the results of a single injection of autologous ADSCs for OA treatment in dogs. Two of them used concentrated ADSCs, which showed a significant improvement in all the scores [46], and produced better results than a control group randomly assigned to placebo and blindly evaluated [47]. A further study used expanded cells, combined with either PRP or hyaluronic acid (HA), and reported improved and comparable outcomes 1 month after the injection [48].

Regarding applications in humans, 10 out of the 11 clinical studies reported the use of nonexpanded autologous ADSCs, as SVF. Adipose tissue was obtained by liposuction from the abdominal area or buttocks in all cases, except for two studies where infrapatellar fad pad tissue was harvested during knee arthroscopy [19, 49]. However, the same authors concluded that more ADSCs can be obtained from the buttocks than from infrapatellar fad pad, with the same differentiation potential in both sources [49].

All except three of these clinical papers described ADSCs injected in varying volumes (3–5 cc) of autologous PRP, activated with Ca-chloride. Three studies used HA as a carrier instead [20, 21, 50], and one of them also added dexamethasone to the cell-PRP-HA mixture [50]. After a single injection of ADSCs, a variable number (usually 2) of PRP-only intra-articular injections were used in most of the studies. In four of these studies cells were injected following arthroscopic lavage [51] and debridement [19, 49, 52–54].

All clinical studies showed that the administration of SVF containing ADSCs improves pain and functional scores at a follow-up of between 3 and 36 months. In three studies MRI analysis was performed, which revealed improved features, including increased cartilage thickness [49, 50, 55]. Four studies evaluated the implant through second-look arthroscopy 6 [36], 12, and 24 months [51, 53, 54] after treatment, which showed maintained or improved joint status in OA patients. However, only one of them performed a histological analysis of the repair tissue, which showed hyaline-like features [55]. Interestingly, the latter study evaluated the effect of different doses of ADSCs and found clinical and histologic improvement and no adverse events only with high-dose administration. Koh and Choi performed the only comparative studies, by testing the effect of ADSCs-PRP (followed by 2 PRP-only injections) with respect to control groups treated with injections of PRP alone [19]. A further study by the same group suggested the beneficial effect of adding fibrin glue to the cells [54].

A preliminary study showed the safety of SVF injections and a significant clinical improvement in both groups [19]. However, no significant difference was found in the outcome, even though the study group (SVF) had significantly lower basal scores [19]. More recently, the same group reported a similar prospective comparison in a group of patients with unicompartmental OA, treated with high tibial osteotomy and randomly assigned to PRP alone or PRP plus SVF injections [53]. At 24-month follow-up, the PRP-SVF group had a better clinical outcome in all KOOS parameters. Whereas statistical significance was reached only in isolated KOOS subgroups, better repair tissue was observed by second look arthroscopy in patients treated with PRP-SVF [53]. A further study on 30 patients found a significant clinical improvement between 12 and 24 months after treatment [51], thus suggesting that the beneficial effect of ADSCs within SVF may be long-lasting. A more recent study used SVF for the treatment of isolated chondral lesions of the knee, as proposed by Koga et al. [56]. Interestingly, the overall significant improvement in terms of clinical scores was negatively affected by higher body mass index (BMI) and larger defect size [52]. Moreover, second look arthroscopic evaluations, performed 1 year after surgery, showed abnormal repair tissue in 76% of the cases; thus, the authors suggested that the use of scaffolds might further improve the quality of the repair tissue [52]. A partial answer to this question came from a very recent study, where patients with isolated cartilage defects in OA knees were treated with the implantation of ADSCs loaded into fibrin glue (FG) as a scaffold [54]. The clinical and macroscopic outcomes at short-term follow-up were compared with those of patients treated with SVF implantation without scaffold. Besides a comparable clinical and macroscopic improvement in both groups, FG significantly improved the tissue quality (ICRS score) by second look arthroscopy [54]. Finally, higher BMI and larger lesions were confirmed to be negative prognostic factors for the treatment of isolated articular surface defects [54]. Despite the new data arising from their studies, the authors concluded that key concerns, such as the best amount of cells and the ideal scaffold to be used, still remain unsolved.

All the clinical studies cited above are case series, with several limitations, and heterogeneity in the methods is the main confounding factor in this review. First, no data have been reported concerning the effects of pure SVF-ADSCs injections and since most studies report SVF administered in a volume of PRP, it is impossible to distinguish the effects of ADSCs isolated from those of PRP or other substances [19], which have already been shown to be effective in OA patients [2, 57]. Similarly, combination with surgical treatments, such as lavage and debridement, provides pain relief in the short-term [52] and requires further examination. Furthermore, no protocol has yet emerged as the most effective in terms of times and modality of administration. Finally, most of these studies lack a control group, whereas randomized, double blind, and placebo-controlled studies would be key to confirming the efficacy of ADSCs in knee joint disease.

## 7. Conclusion

In conclusion, conversely to the tendency found in the preclinical setting, the injective intra-articular delivery of SVF has emerged as the trend in clinical use. In fact, safety, feasibility, and effectiveness of this approach have been demonstrated at preclinical level, and several aspects favor the use of freshly harvested SVF instead of expanded ADSCs. Concentrating ADSCs would obviate the high costs related to cell manipulation and culture, thus exploiting the increased vitality and differentiation attitude of cells. Moreover, a minimally invasive procedure as injective delivery is more readily accepted by patients due to lower rates of morbidity and adverse events. Furthermore, although the ideal requirement for maximizing the regenerative effect of such therapies would suggest the use of processed cells, there is still lack of consensus about the most effective ADSCs dosage. Currently, the lack of well-designed and comparative studies focusing on the different procedures still leaves many questions unanswered about the mechanism of action of ADSCs, the best way to apply them, and their real therapeutic potential.

## Figures and Tables

**Table 1:** Preclinical studies on animal model.

	Animal model	Defect type	Delivery method	ADSCs processing	Study design	Results
Nathan et al. 2003, *Tissue Engineering* [22]	Rabbit	Osteochondral defect	Surgical implantation	Expanded homologous	(i) ADSCs + fibrin (ii) Periosteum MSCs + fibrin (iii) Fibrin only	Better defect reconstruction in experimental groups than in control, with superior results for ADSCs

Masuoka et al. 2006, *J Biomed Mater Res * [41]	Rabbit	Osteochondral defect	Surgical implantation	Expanded autologous	(i) ADSCs + ACHMS-scaffold (ii) ACHMS-scaffold alone (iii) Empty defect	New regenerated hyaline-like cartilage only in the ADSCs group

Dragoo et al. 2007, *Tissue Eng* [36]	Rabbit	Osteochondral defect	Surgical implantation	Expanded autologous	(i) ADSCs + fibrin glue matrix (ii) Empty defect	100% of defects healed in ADSCs group and only 8% in control group

Koga et al. 2008, *Cell Tissue Res* [24]	Rabbit	Osteochondral defect	Surgical implantation	Expanded homologous	(i) ADSCs (ii) BMSCs (iii) Synovium MSCs (iv) Muscle MSCs	Great amount of cartilage matrix production in BMSCs and synovium MSCs compared to other groups Low chondrogenic potential for ADSCs

Zhang et al. 2009, *Chin J Traumatol* [40]	Rabbit	Osteochondral defect	Surgical implantation	Expanded homologous	(i) ADSCs + calcium alginate gel (ii) Calcium alginate gel alone (iii) Empty defect	Complete defect healing only in the ADSCs group

Frisbie et al. 2009, *J Orthop Res* [27]	Horse	Induced OA (by arthroscopy)	Injective	Expanded autologous	(i) ADSCs (ii) BMSCs (iii) Placebo (sham surgery)	No adverse treatment-related events ADSCs decreased synovial fluid PDE G2 Overall improvement with cells n.s.

Cui et al. 2009, *Biomaterials* [37]	Pig	Osteochondral defect	Surgical implantation	Expanded autologous chondrogenic-induced	(i) ADSCs + PGA/PLA (ii) PGA/PLA alone	Hyaline-like neo-cartilage Well-integrated implant Lack of cartilage repair for acellular group

Oliveira et al. 2010, *J Orthop Res* [45]	Rabbit	Osteochondral defect	Surgical implantation	Expanded autologous chondrogenic-induced	(i) Predifferentiated ADSCs + gellan gum (GG) (ii) Not predifferentiated ADSCs + GG (iii) Articular chondrocytes + GG (iv) GG alone (v) Empty defect	Good defect filling and integration with surrounding cartilage for cell-loaded constructs Best results with predifferentiated ADSCs with GG

Im and Lee 2010, J *Biomed Mater Res B Appl Biomater* [23]	Rabbit	Osteochondral defect	Surgical implantation	Expanded autologous	(i) ADSCs + (PCL)/F127 scaffold (ii) Scaffold alone (iii) TGF-b2 + BMP-7 + scaffold (iv) ADSCs + TGF-b2 + BMP-7 + scaffold	Incomplete cartilage healing for all groups Better macroscopic score in growth factors groups compared to ADSCs alone

Li et al. 2011, *Artif Cells Blood Substit Immobil Biotechno*l [25]	Rabbit	Osteochondral defect	Surgical implantation	Expanded autologous	(i) ADSCs + DBM (ii) BMSCs + DBM (iii) Synovium MSCs + DBM (iv) Periosteum MSCs + DBM (v) Muscle MSCs + DBM (vi) Empty defect	Good defect filling with new hyaline-like cartilage for each treatment group BMSCs had best results for cartilage matrix synthesis, gross morphology, and histology

Toghraie et al. 2011, *The Knee* [32]	Rabbit	Induced OA ACL transection (ACLT)	Injective	Expanded homologous	(i) ADSCs from infrapatellar fat pad (ii) Control (medium only)	Better cartilage quality, lower subchondral changes, and lower OA features for ADSCs group than controls

Toghraie et al. 2012, *Arch Iran Med* [33]	Rabbit	Induced OA (ACLT)	Injective	Expanded homologous	(i) ADSCs from subcutaneous fat (ii) Control (medium only)	Improved cartilage quality for ADSC group compared to control

Lee and Im 2012, *Biomaterials* [43]	Rat	Induced OA (ACLT)	Injective	Expanded transduced SOX-trio genes autologous	(i) ADSCs transduced with SOX-trio genes + fibrin gel (ii) ADSCs transfected with empty vector (iii) Fibrin gel alone	Decrease of OA progression Decrease of OA progression for ADSCs in fibrin gel group
			Surgical implantation			Good hyaline cartilage-like tissue regeneration for the transfected ADSCs/fibrin gel group Incomplete healing and fibrous tissue in both controls

Ter Huurne et al. 2012, *Arthritis Rheum* [34]	Mouse	Induced OA (collagenase injection)	Injective	Expanded autologous	(i) ADSCs + 4% mouse albumin (ii) Only 4% mouse albumin	Inhibition of OA progression in early stage of OA, whereas no effects with injections in late OA stage

Xie et al. 2012, *Biomaterials* [44]	Rabbit	Osteochondral defect	Surgical implantation	Expanded homologous	(i) ADSCs + PRP (ii) BMSCs + PRP (iii) PRP alone (iv) Empty defect (v) Sham treatment	Better repair for cell + PRP groups versus PRP alone BMSCs higher chondrogenic differentiation ADSCs differentiate into mature chondrocytes

Cao et al. 2012, *J Biomed Biotechnol* [58]	Rabbit	Osteochondral defect	Surgical implantation	Expanded autologous	(i) ADSCs-bBMPs-bilayered construct (ii) bBMPs-bilayered construct (iii) Empty defect	Better defect healing and integration with host tissue in the ADSCs-bBMPs-bilayered construct group

Desando et al. 2013, *Arthritis Res Ther* [35]	Rabbit	Induced OA (ACLT)	Injective	Expanded autologous	(i) 2 mln ADSCs + 4% rabbit serum (ii) 6 mln ADSCs + 4% rabbit serum (iii) 4% rabbit serum alone (iv) No treatment (v) Sham group	Decrease of OA progression in the study groups compared to controls Best results for implanted 2 mln ADSCs

van Pham et al. 2013, *Stem Cell Res Ther* [29]	Mouse	Induced OA (needle disruption)	Injective	Expanded human ADSCs	(i) ADSCS + 15% PRP (ii) ADSCS + 10% FBS (iii) PBS only (iv) No treatment, no defect	Tendency for better cartilage repair improvement for cell-transplanted groups compared to PBS group, significantly better results for PRP-pretreated ADSCs on all parameters

van Pham et al. 2013, *J Med Engineering* [30]	Mouse	Induced OA (needle disruption)	Injective	Human SVF	(i) ADSCs + PRP (ii) Only PBS (iii) No treatment, no defect	Significant improvement in articular cartilage regeneration for PRP-pretreated ADSCs compared to PBS control No adverse events, high safety

Jurgens et al. 2013, *Bio Research* [31]	Goat	Osteochondral defect	Surgical implantation	Expanded versus SVF autologous	(i) Expanded ADSCs + collagen I/III (ii) Concentrated ADSCs + collagen I/III (iii) Collagen I/III alone	Good tissue regeneration in all groups with tendency towards better results in cell-treated groups and in particular for concentrated ADSCs No adverse events

Zhang et al. 2013, *Acta Biomaterialia* [39]	Rabbit	Osteochondral defect	Surgical implantation	Expanded chondrogenic-induced autologous	(i) Predifferentiated ADSCs + PLGA/CHI (ii) PLGA/CHI alone	Good tissue regeneration, similar to healthy cartilage, well integrated with surrounding tissue in ADSCs/scaffold group compared to scaffold alone

Shi et al. 2013, *Arthroscopy* [42]	Rabbit	Osteochondral defect	Surgical implantation	Expanded and transfected autologous	(i) BMP-4 transfected ADSCs + PLLGA (ii) Nontransfected ADSCs + PLLGA (iii) PLLGA alone	Better improvement in chondrogenesis *in vivo* for the transfected ADSCs group with respect to untransfected and scaffold alone

Gong et al. 2014, *Tissue Eng part A* [38]	Pig	Osteochondral defect	Surgical implantation	Expanded chondrogenic-induced autologous	(i) ADSCs + PGA/PLA (ii) PGA/PLA alone	Successful new cartilage Graft remodeling between 3 and 6 m Proteomic analysis detected differences versus native cartilage

Wang et al. 2014, *Genet Mol Res* [59]	Rabbit	Osteochondral defect	Surgical implantation	Expanded homologous	(i) ADSCs preseeded ACM (ii) ACM only (iii) Empty defect	Chondral tissue with hyaline features in ADSC-ACM group, with type II coll., fibrous tissue in ACM only

Ude et al. 2014, *PLoS One* [26]	Sheep	Induced OA (ACLT)	Injective	Expanded chondrogenic-induced autologous	(i) ADSCs (ii) BMSCs (iii) Control	ADSCs had higher cell proliferation and BMSCs had better chondrogenic inductions and gene expressions ICRS score n.s. ADSCs versus BMSCs

**(a):** 

Ref.	Study type	Animal	Pathology	Cell type and source	Injection/implantation	Study design	Follow-up	Results
Black et al. 2007, *Vet Ther* [47]	Randomized blinded placebo-controlled	Dogs	OA	Concentrated ADSCs, from abdominal area, inguinal, or thoracic wall regions	1 injection	(i) ADSCs (ii) placebo	90 days	Significantly improved scores for lameness and the compiled scores for lameness, pain, and range of motion compared with control dogs

Black et al. 2008, *Vet Ther* [46]	Case series	Dogs	OA	Concentrated ADSCs, from abdominal area, inguinal, or thoracic wall regions	1 injection	(i) ADSCs	180 days	40% improvement in lameness, range of motion, and pain on manipulation over time compared with baseline values

Guercio et al. 2012, *Cell biology international* [48]	Comparative	Dogs	OA	Expanded, from abdominal area, subcutaneous, visceral, and inguinal fat depots	1 injection	(i) ADSCs + PRP (ii) ADSCs + HA	1 month	Both groups showed functional improvements in their disability, lameness on trotting, and pain on manipulation of the joints

**(b):** 

Ref.	Study type	Pathology	Cell type and source	Injection/implantation	Study design	Number of patients	Follow-up	Results
Pak 2011, *J Med Case Reports* [50]	Case report	Knee OA	SVF Abdominal area	1 injection	(i) ADSCs + PRP + HA + dexamethasone	2	3 months	Cartilage volume increased at MRI Both improved function

Koh and Choi 2012, *The Knee* [19]	Comparative study	Knee OA	SVF Infrapatellar fad pad	1 injection after debridement	(i) ADSCs + PRP (ii) Only PRP (control)	Study group: 25 Control group: 25	16.4 months	Significant improvement in all clinical scores Study versus control: n.s. at final follow-up, but study group had lower basal

Pak et al. 2013, *BMC Musculoskeletal Disorders* [20]	Case series	Knee OA	SVF Abdominal area	1 injection	(i) ADSCs + PRP + HA	91	30 months	VAS improved 50–60% No major complications

Pak et al. 2013, *PLoS One* [21]	Case series	Chondromalacia patellae	SVF Abdominal area	1 injection	(i) ADSCs + PRP + HA	3	12 months	Pain improved: 50–70% at 1 m 80–90% at 3 m

Koh et al. 2013, *Knee Surg Sports Traumatol Arthrosc* [51]	Case series	Knee OA	SVF Buttocks	1 injection	(i) ADSCs + PRP	30	24 months	Significant clinical improvement 14/16 (87.5%) of 2nd look arthroscopy within 24 m improved or maintained cartilage status Further clinical improvement 24 versus 12 m

Koh et al. 2013, *Arthroscopy* [49]	Case series	Knee OA	SVF Infrapatellar fad pad	1 injection after debridement	(i) ADSCs + PRP	18	24.3 months	Significant improvement of the clinical and MRI scores at final follow-up

Bui et al. 2014, *Biomedical Research and Therapy* [55]	Case series	Knee OA	SVF Abdominal area	1 injection	(i) ADSCs + PRP	21	6 months	Function improvement in all patients at 8.5 m. Increased cartilage thickness on MRI

Jo et al. 2014, *Stem Cells* [60]	Case series	Knee OA	Expanded Abdominal area	1 injection	(i) Phase I: 3 doses of ADSCs; the low-, mid-, and high-dose group with 3 patients each (ii) Phase II: 9 patients receiving the high dose of ADSCs	18	6 months	Clinical improvement and hyaline-like regenerative tissue only in high-dose group, without adverse events

Koh et al. 2014, *Am J Sports Med* [52]	Case series	Knee OA	SVF Buttocks	1 injection after debridement	ADSCs local adherent technique	35 (37 knees)	12.7 months	Clinical improvement; 94% patients excellent or good satisfaction 76% abnormal or severely abnormal repair tissue at 2nd look

Koh et al. 2014, *Arthroscopy* [53]	Comparative study	Knee OA	SVF Buttocks	1 injection after debridement	(i) HTO + PRP + ADSCs (*n* = 23) (ii) HTO + PRP (*n* = 21)	44	24 months	Better clinical improvement in PRP + ADSCs group (some KOOS subgroups) Better tissue healing at 2nd look for PRP + ADSCs

Kim et al. 2015, *Am J Sports Med* [54]	Comparative study	Knee OA	SVF Buttocks	1 injection after debridement	(i) ADSCs local adherent (*n* = 37) (ii) ADSCs on FG (*n* = 17)	54 (56 knees) second look	28.6 m 12.3 m (second look)	Overall clinical improvement Comparable for both groups Better ICRS scores at 2nd look for ADSC-FG group Lower BMI and smaller size positively correlate with outcomes
